# Development of a Check-All-That-Apply (CATA) Ballot and Machine Learning for Generation Z Consumers for Innovative Traditional Food

**DOI:** 10.3390/foods11162409

**Published:** 2022-08-11

**Authors:** Bo Wang, Che Shen, Ting Zhao, Xiuwen Zhai, Meiqi Ding, Limei Dai, Shengmei Gai, Dengyong Liu

**Affiliations:** 1 College of Food Science and Technology, Bohai University, Jinzhou 121013, China; 2 Faculty of Educational Studies, University Putra Malaysia, Seri Kembangan 62502, Malaysia; 3 Jiangsu Collaborative Innovation Center of Meat Production and Processing, Quality and Safety Control, Nanjing 210095, China

**Keywords:** Check-All-That-Apply, generation Z, machine learning, grounded theory, lamb shashlik, cooking method

## Abstract

Generation Z (Gen Z) consumers account for an increasing proportion of the food market. The aim of this study took lamb shashliks as an example and developed novel products from the perspective of cooking methods in order to develop a traditional food suitable for Gen Z consumers. The sensory characterization of electric heating air (EH), microwave heating (MH), air frying (AF), and control (traditional burning charcoal (BC) of lamb shashliks) was performed using the CATA methodology with 120 Gen Z consumers as assessors. A 9-point hedonic scale was used to evaluate Gen Z consumers’ preferences for the cooking method, as well as a CATA ballot with 46 attributes which described the sensory characteristics of lamb shashliks. The machine learning algorithms were used to identify consumer preferences for different cooking methods of lamb shashliks as a function of sensory attributes and assessed the relationship between products and attributes present in the perceptual map for the degree of association. Meanwhile, sensory attributes as important variables play a relatively more important role in each cooking method. The most important variables for sensory attributes of lamb shashliks using BC are char-grilled aroma and smoky flavor. Similarly, the most important variables for AF samples are butter aroma, intensity aroma, and intensity aftertaste, the most important variables for EH samples are dry texture and hard texture, and the most important variables for MH samples are light color regarding external appearance and lumpy on chewing texture. The interviews were conducted with Gen Z consumers to investigate why they prefer innovative products—AF. Grounded theory and the social network analysis (SNA) method were utilized to explore why consumers chose AF, demonstrating that Gen Z consumers who had previously tasted AF lamb shashliks could easily perceive the buttery aroma. This study provides a theoretical and practical basis for developing lamb shashliks tailored to Gen Z consumers.

## 1. Introduction

Members of Gen Z, also known as post-millennials, were born between 1995 and 2009 [1]. As the world’s largest consumer group, Gen Z’s influence and purchase ability facilitate the upgrading of consumption [2]. In particular, their diverse needs and creativity with food have actively upgraded consumption in the tourism and catering industries, and their psychological characteristics, consumer perceptions, consumption behaviors, and consumer preferences affect the business trends of food companies. Gen Z is an increasingly important consumer segment in the food market, significantly impacting food purchases [3]. Gen Z in China comprises approximately 260 million people, accounting for 19% of the total population [4]. China has the world’s largest population of Gen Z, and this demographic is becoming the country’s primary consumers [5].

As modern food enthusiasts, Gen Z finds and shops for the latest products, ingredients, and restaurants mainly for food that brings a “good impression” [6]. Education and urbanization in China have led to Gen Z being eager for a higher quality of life, paying more attention to the environment, and putting health first when making food choices [2,7]. Therefore, based on this concept, it is essential to develop a product that meets the sensory expectations of Gen Z consumers while also creating healthy and environmentally friendly products.

Many traditional foods are popular with many consumers due to their unique flavors, such as lamb shashliks, which have unique sensory characteristics [8,9,10,11]. The disadvantage of this traditional food is that the fumes emitted during the preparation process pollute the environment. In particular, the smoke from fuel oil contains a lot of P.M_2.5_ and organic aerosols [12,13]. The innovation of traditional food is not only to be processed in traditional ways or according to traditional recipes, but also to ensure the sensory properties of the product, so as to be recognized by consumers [14]. At the same time, promising goals that affect the meat consumption behavior patterns of Gen Z consumers include the coordination of environmental and health information, a reference framework for healthy and sustainable eating, and attitudes, values, subjective norms, cooking styles, habits, and traditions [15,16,17]. Therefore, this study develops lamb shashliks suitable for Gen Z consumers from the perspective of cooking method.

Technological developments have produced other cooking methods, such as electric heating air (EH), microwave heating (MH), and air frying (AF) that can avoid the disadvantages of traditional lamb shashliks while still releasing the fragrances of the meat [18,19,20,21,22,23].

Although the JAR scale is a popular method of collecting actionable information for product development, Ares et al. [24] indicated that the JAR scale may be challenging for consumers because they need to simultaneously evaluate the perceptual strength of sensory attributes, the intensity of ideal attributes, and the differences between the perceptual intensity and ideal intensity. The JAR scales make consumers focus their attention on specific sensory attributes, which may increase awareness of how the product falls short of the ideal and ultimately change their hedonic perception of the sample.

The CATA (Check-All-That-Apply) method is a technique that enables sensory analysis of food by selecting attributes presented in a questionnaire and is considered more accessible and faster than the use of well-trained assessors [25]. Dooley et al. [26] suggested that this may be a more practical approach from a consumer-led product development perspective. CATA methodology provides information on what attributes consumers can detect and how this might relate to their overall preferences and acceptance. In this methodology, consumers are presented with a range of sensory terms and asked to choose all of the terms they think are appropriate to describe the focal sample. It is reported that this method is simple and intuitive for consumers and provides practical and repeatable product information [26,27,28]. Furthermore, CATA questions are not likely to influence consumers’ hedonic responses [29,30]. 

Assessing consumers’ food preferences, acceptability, and choices is the most critical task in the food industry’s product decision-making process. Consumer research is a complicated and time-consuming process, and the ability to predict overall preferences is crucial in market research [31]. The machine learning method assumes that the observed data are generated by an unknown and complex process and relies on an algorithm to learn the pattern and predict the response to an independent variable [32]. Software development has increased the popularity of such methods in recent years [33]. However, machine learning has not been widely used in analyzing sensory and consumer preference data or meat science.

The main goal of this work was to develop novel lamb shashliks for Gen Z consumers using the following: (1) the sensory characterization of control (BC), AF, EH, and MH lamb shashliks using the CATA methodology, (2) the machine learning algorithms to identify consumers’ preferences and important variables for lamb shashliks with four different cooking methods, and (3) investigating why they prefer innovative lamb shashliks using grounded theory and SNA.

## 2. Materials and Methods 

### 2.1. Meat Preparation

Out of 120 available, 24 six-month-old Sunite sheep (a Chinese breed) grazing together in the Inner Mongolia Autonomous Region, China, with a similar genetic background and the same diet. The longest section of spine lean meat and tail fat were removed after slaughter in accordance with the Chinese Standard Agreement GB 2707-2016. Carcasses were aged for 48 h at 0–4 °C. Meat and tail fat were frozen at −20 °C and transported to Jinzhou, Liaoning, China, using typical cold chain logistics.

The meat and fat were thawed in a 4 ± 1 °C incubator (Panasonic MIR-154-PC, Panasonic (China), Beijing, China) for approximately 8 h until the core temperature reached −3 to −5 °C. After removing surface fat, the lean and fatty meat was cut into approximately 2 cm 3 cubes (2 × 1 × 1) according to the “Cutting Technical Specification of Lamb of the Ministry of Agriculture and Rural Affairs of the People’s Republic of China” (NY/T 1564-2007). A 15 cm bamboo stick was used to skewer four pieces of lean meat and one piece of fat, and each piece of meat was 5 g, for a total of 25 g.

### 2.2. Cooking Methods

An end temperature of 80 °C was set as the internal temperature at which the cooking process was stopped, and measured with a precision thermometer (Reference Benetech^®^, Benetech Instrument, Shenzhen, China). BC cooking: the lamb shashliks were grilled for 12 min on the stove 10 cm above the charcoal at a temperature of 220–230 °C and turned every 1 min. EH cooking: the lamb shashliks were placed in an electric oven (Panasonic NB-HM3810, Panasonic (China), Beijing, China) at 200 °C for 10 min and turned every 1 min. MH cooking: the lamb was placed in a 700 W microwave oven (Galanz G70F23CN2P-BM1S0, Galanz, Foshan, China) for 8 min at 2450 MHz and turned every 1 min. AF cooking: the lamb shashliks were placed in an air fryer (Midea KZ120Q7-400G, Midea, Foshan, China) at 1700 W for 5 min every 30 s.

### 2.3. Design of CATA Ballot

#### 2.3.1. Focus Groups

CATA terms for grilled lamb shashliks were generated by consumers not testing the product, i.e., focus groups. In total, four focus groups with five Gen Z members each were formed, for a total of 20 people. Members were selected based on gender (male and female) and age (18–26 years). The consumers participating in the focus groups were recruited from the consumer database of Bohai University. The participants were in line with the age characteristics of Gen Z and met the consumption requirements of having eaten barbecue within a week and liking barbecue. Each participant authorized this study. 

Focus group discussions took place in the conference room of Bohai University and lasted approximately 30 min. All meetings were hosted by the same experienced researcher (one of the first authors of this article) to ensure consistency of the interview style [34]. The moderator handled the video recording and critical notes about participants’ behavior with the assistance of other co-authors.

Focus group guidelines were based on Rose’s work [35]. After an introduction and icebreaker, the session was divided into four phases: (i) exercise context—participants were asked to describe a piece of traditional Chinese spiced beef, specifically “How does it look?” and “What do you think is the most important of 1 to 9?”; (ii) food exercise—participants were asked to describe the appearance, aroma, and flavor of the Chinese spiced beef, then asked to choose the best word to describe the traditional Chinese spiced beef; (iii) product description—participants were asked to describe samples of lamb shashliks prepared using BC (appearance, aroma, texture, flavor, and aftertaste), then to choose which features were most important; and (iv) product description—participants were asked to describe samples of lamb shashliks prepared using EH, AF, and MH (appearance, aroma, texture, flavor, and aftertaste), then to choose which characteristics were most significant for each preparation method. Eventually, the focus group found the forty-six attributes that would be evaluated.

The focus group was videotaped (subject to the informed consent of the participants) to ensure the accuracy of transcription and analysis. All recordings were transcribed verbatim and anonymously.

#### 2.3.2. Data Analysis

Transcriptions of the focus groups discussions were analyzed, and core themes were formulated based on the data analysis, considering the similarities and differences in participants’ responses. In this study, the core themes were the sensory dimensions and the perceptual characteristics of Gen Z consumers within each dimension.

Due to the similarity of terms, a common set of descriptors was created for all four lamb shashliks in the questionnaire to ensure a consistency of responses. 

Data analysis was performed using XLSTAT 2019 (https://www.xlstat.com/en/ accessed on 20 June 2021) and SPSS 26.0 software (IBM Corporation, Armonk, NY, USA).

### 2.4. Evaluation of Samples

#### 2.4.1. Sensory Evaluation

Consumers from the consumer database of Bohai University were invited to participate via WeChat and Tencent QQ (participants different from focus groups). Recruitment criteria were the following: age (18–26 years old), culture (Chinese), and barbecue consumption frequency (at least once per month). A total of 120 Chinese Gen Z consumers were recruited to evaluate the lamb shashliks for 4 cooking methods. The average age was 22.6 (±1.8) years, and 62% were female.

Participants were assigned a random code to ensure anonymity. The experimental procedure of each phase was explained, and a written consent form indicating voluntary participation was signed by all participants prior to beginning the study.

A 9-point hedonic scale, ranging from 1−“dislike extremely” to 9−“like extremely”, was used to describe the overall liking of the lamb shashlik samples of the four cooking methods under evaluation [36]. According to Michael Meyners and John C. Castura [37], a “to assessor” scheme was employed, where CATA attributes were assigned to assessors such that each assessor had a specific fixed list of attributes that repeats, but each assessor had a different list order. This freed up assessor cognitive capabilities for a more comprehensive product evaluation.

Tests were carried out in the sensory evaluation laboratory of Bohai University. The tasting room complied with the standard ISO 8589: 2007-Sensory Analysis: General Guidelines for the Design of Test Room. The laboratory was equipped with ten isolated sensory booths that had white tables, each accommodating one person, and was illuminated with white light. The prepared lamb shashliks were put into thermal insulation bags, sorted according to the cooking method, and then put into the thermal insulation box. At the beginning of sensory evaluation, the researchers put the samples into white sensory cups. Samples were presented randomly, one at a time, in a continuous series in a single session. Each sample was in a white sensory cup coded with a random three-digit number. The general procedures and attributes of the CATA ballot were explained by an experienced researcher. Each participant received four samples of lamb shashliks. Four lamb shashliks were randomly provided to ensure that participants had sufficient samples to perform liking and CATA tasks.

Samples were presented in a single balanced order to offset possible carry-over effects in accordance with the Latin square design [38]. Participants were provided with a spittoon, a glass of bottled natural purified water, and salt-free biscuits and were asked to chew a biscuit and rinse their mouths with water between samples to cleanse their palate. The tasting was carried out under the supervision of researchers and all participants were willing to follow the test instructions without any significant difficulties.

#### 2.4.2. Data Analysis

The results of overall liking were evaluated with descriptive statistics (mean and standard error of the mean) and with a one-way ANOVA, where cooking methods were used as main factors, followed by the LSD (Least Significant Difference) comparison test. The pairwise interactions of cooking methods were also evaluated at a 95% confidence level.

To analyze CATA questions, frequency of use of each term was determined by counting the number of consumers who used each attribute to describe the samples, and a Cochran Q test at a 95% confidence level was initially used to identify significant differences perceived by consumers between samples for each of the terms [39].

The frequency of each term used in the CATA ballot was determined by calculating the number of consumers using each sample attribute description. The 95% confidence level Cochran’s Q test was used to identify significant differences between consumers’ use of each term [39]. To obtain a two-dimensional representation of each sample, correspondence analysis (CA) was applied to the previously determined contingency table. This analysis provided a sensory map of the sample that could be used to determine the similarities and differences between samples and their characteristic properties [40]. 

### 2.5. Machine Learning

Based on the CATA ballot results of Gen Z consumers, machine learning techniques were used, including support vector machines (SVM), gradient boosting decision tree (GBDT), deep neural networks (DNN), and a novel model, orthogonal matching pursuit—stochastic gradient descent (OMP-SGD).

For SVM, the selection process of kernel function and penalty function is considered: (i) first, a penalty function C is randomly fixed, and the function with the best result is found from “linear kernel function”, “polynomial kernel function”, and “radial basis kernel function (Gaussian kernel function)” and applied; (ii) second, the optimal kernel function is fixed, and the penalty function with the best result from “0.1”, “0.5”, “1”, “3”, and “5” is found and applied.

For GBDT, two randomization processes are considered: (i) first, a guide sample is obtained from the learning set of each tree, (ii) then, a subset of explanatory variables is randomly selected at each node.

OMP-SGD combines the advantages of OMP and SGD to build a single model to optimize prediction performance [41,42,43]. The operation of the algorithm is as follows: firstly, the initial matrix of OMP is established, and then the SGD, with the goal of reducing the residual value of OMP established. Finally, a model with high accuracy of the prediction results is obtained through many iterations.

DNN consists of input layer, hidden layer, and output layer, and the neurons of the two adjacent layers are connected. All weights of the neural network are randomly generated based on Gaussian distribution, and the activation function between layers is composed of 0.2x scaled LeakyReLU. The loss function adopts MAE, and the optimization function uses the Adam algorithm with 0.001 learning rate, and the BatchSize is 32. Although the parameters are not adjusted, DNN still has a very fast convergence rate.

Each model (using Python 3.7, JetBrains, Bragg, Czech Republic) divided data into 70% for model development and 30% for verification, using overall preference as the dependent variable and sensory descriptor as the independent variable.

### 2.6. After Evaluation

#### 2.6.1. Interview

Based on the results of the CATA ballot, an informal interview was conducted with participants, asking which cooking method they considered to be unique and why, as well as their reasons for not choosing the other three. Out of 120 Gen Z consumers, 10 people were randomly selected, and each informal interview lasted 5 min. Eight people reported that a buttery aroma is the primary reason for their choosing AF. However, a small number of people cannot smell this aroma. Lamb contains the ingredients of a buttery aroma [44,45]; however, not all the relevant studies demonstrate that consumers can detect such an aroma, despite it being present. It was, therefore, necessary to conduct formal interviews to elucidate this phenomenon.

Researchers interviewed Gen Z consumers who could smell the buttery aroma. Appointments were made by telephone, QQ, and WeChat, and participants were informed of the subject and content of the interview in advance. Twenty-nine randomly selected interviewees were invited, of which fourteen were male (48.3%) and fifteen were female (51.7%). One-on-one interviews lasting approximately 30 min were conducted in the conference room of Bohai University. The same experienced researcher conducted all interviews to ensure consistency of interview style. Interviews were recorded with the consent of the interviewees and were transcribed. Interviewee numbers (1–29) were used to identify files.

The interview questions incorporated experimental findings and proposed four hypotheses addressing users’ attributes and psychological perspectives to determine the factors that affect consumers’ experience of a buttery aroma: (i) the aroma was very rich a few minutes before it peaked, but its duration was short, (ii) the aroma could be perceived but there was almost no buttery flavor when tasting, (iii) perception of the aroma was related to age, gender, preference for sweets or barbecue, and whether the participant had previously tasted AF products, (iv) if the surroundings influenced aroma perception during the sensory evaluation and if it was easy to be hinted at psychologically.

Interviews were in two parts: respondents’ basic information followed by the main content. Basic information included whether participants had eaten barbecue in the past week, if they liked barbecue and sweets, if they had eaten sweets in the past three days, if they had previously eaten food from an air fryer, how they felt during the sensory evaluation process (happy, normal, or unhappy), their gender and age, and if they had experienced rhinitis or other diseases that affect the sense of smell. The main interview content covered was: (i) whether the buttery aroma could be compared with other foods, (ii) a description of the process of smelling and tasting, (iii) if the buttery aroma could be perceived in cooking methods other than AF (if the interviewee perceived the buttery aroma, they were asked to describe the experience, but if they could not smell it, they were asked to analyze the reasons from their point of view), (iv) if they would continue to eat grilled AF lamb shashliks in the future, and (v) participants’ opinions of why AF lamb shashliks had a buttery aroma and what their expectations were for its future development.

#### 2.6.2. Data Analysis

According to the interview results of Gen Z consumers, the grounded theory research approach was adopted to explore why Gen Z consumers prefer this product, so as to ensure that consumer experiences are more targeted and persuasive. Grounded theory [46] refers to collecting and encoding data concerning a specific social phenomenon. Coding is the process of decomposing, discriminating, and conceptualizing the empirical data. Concepts and categories are summarized and refined from the original data. Constant comparison and dialectical correction are required throughout the analytical process. Finally, a new theory that is rooted in actual data and reflects the essence of the phenomenon is formed. Such an approach is suitable for elucidating the reasons why consumers prefer a particular product.

The perception of buttery aroma is affected by interactions among the relevant factors, while traditional statistical methods have many limitations. The multi-factor dimensionality reduction (MDR) method proposed by Ritchie et al. [47] overcomes some of these shortcomings to a certain extent, as it can analyze interactions between high-dimension data from limited sample sizes using the MDR 3.0.2 software package. Standardizing the organized text data from the interviews and importing it into NVivo 11 plus software introduced a grounded theory method for coding the text data level-by-level and generating nodes. The coded interview materials used social network analysis (SNA) and UCINET analysis tools to visualize the network. Degree is an essential indicator in SNA that measures the position of a node in a network. The higher the centrality of a node degree, the more the point is in the center of a network. The size of the node and degree of centrality can be obtained by calculation. The thickness of the line between nodes indicates the distance of the relationship.

## 3. Results 

### 3.1. CATA Ballot Development and Overall Liking

The Gen Z consumers elicited a total of 46 sensory attributes for the grilled lamb shashliks, and of the five dimensions (aroma, appearance, flavor, texture, and aftertaste), most attributes were related to flavor (13). 

The finalized CATA ballot is shown in Table 1 and included 46 attributes divided by sensory dimension:7 for aroma (intensity, char-grilled, roast lamb, buttery, liver, oily, fatty), 10 for appearance (caramel on bottom external, light color external, dark external, juicy internal, pink internal, brown internal, juicy external, connective tissue internal, wet, greasy), 13 for flavor (intensity, smoky, roast lamb, liver, bloody, metallic, fatty, gravy, gamey, greasy, bitter, sour, sweet), 8 for texture (tenderness, rubbery, chewy, lumpy on chewing, crumbly, spongy, dry, hard), and 8 for aftertaste (intensity, meaty, liver, bloody, oily, lactic, sour, sweet).

From the one-way ANOVA analysis, a significant effect of different cooking methods (*p* < 0.001) was identified for overall liking (Table 2). According to the LSD (Least Significant Difference) test, Table 3 shows that there are significant differences in consumer preferences when comparing the four products in pairs (*p* < 0.05). BC and AF samples had the higher overall liking scores, whereas EH and MH samples were the least preferred (Table 2).

### 3.2. Sensory Discrimination and Correspondence Analysis

Results yielded the sensory discrimination of the grilled lamb shashliks under the four cooking methods, and Gen Z consumers identified 65.2% of the attributes (30/46) as discriminant. Cochran’s Q test showed significant differences in the frequency with which 30 of the 46 attributes were used to describe the samples of grilled lamb shashliks under the four cooking methods (Table 1). At the aggregate level, the attributes roast lamb aroma, fatty aroma, dark external appearance, brown internal appearance, roast lamb flavor, gamey flavor, chewy texture, dry texture, and meaty aftertaste showed the highest frequency of use when consumers were asked to describe the sensory characteristics of the lamb shashliks under the four cooking methods. These attributes showed an average frequency of use higher than 50%. It is interesting to note that the grilled lamb shashliks under the four cooking methods had significant differences in more attributes of sensory dimensions such as aroma, flavor, appearance, and texture, while most attributes of aftertaste had no difference.

**Figure 1:**
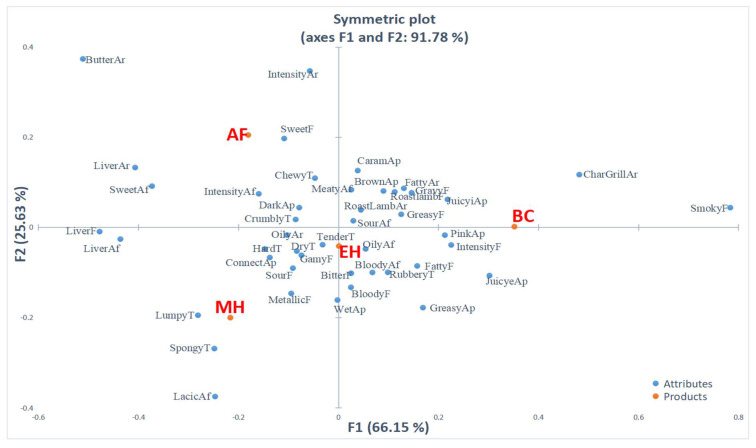
Correspondence analysis of CATA frequencies containing the attributes related to aroma (Ar), appearance (Ap), flavor (F), texture (T), and aftertaste (Af) that significantly differentiated for Gen Z consumers samples (according to the Cochran’s Q test at 95% significance level).

The use of correspondence analysis allows visualization of the contingency table (Table 1) in orthogonal dimensions and illustrates where products under the four cooking methods are located on the map. CA can be considered as a generalization of principle component analysis (PCA) when working with ordinal and nominal data [48]. Figure 1 is a representation of the samples and attributes in the first two coordinates of the CA performed on the frequency table. Combined, the first and second dimensions explained 91.78% of the variance in the data, with the first dimension (66.15%) and second dimension (25.63%). The CA analysis showed that the first dimension was positively correlated with the attributes char grilled aroma and smoky flavor and negatively correlated with the attributes liver flavor and liver aftertaste. The second dimension was positively correlated with the attributes buttery aroma and intensity aroma and negatively correlated with light color external appearance, spongy texture, and lactic aftertaste.

The properties of the four groups were well separated (distance between the samples is a measure of their similarity [49]): BC, located at positive values of the first and second dimension; AF, located at negative values of the first dimension but positive values of the second dimension; EH, located at positive values of the first dimension but negative values of the second dimension; and MH located at negative values of the first and second dimensions. Gen Z consumers associated BC with char grilled aroma and smoky flavor, while they associated AF with buttery aroma and intensity aroma. Similarly, Gen Z consumers associated EH with dry texture and gamy flavor while MH was associated with light color external appearance.

### 3.3. Important Variables

The machine learning methods deep neural network, gradient boosted trees, support vector machine, and orthogonal matching pursuit—stochastic gradient descent were applied to identify the consumer preferences of lamb shashliks of different types as a function of the sensory attributes and evaluated the degree of association between products and attributes present in the perception map. The machine learning methods according to Cochran’s Q test were performed using only the attributes that distinguish samples.

**Figure 2:**
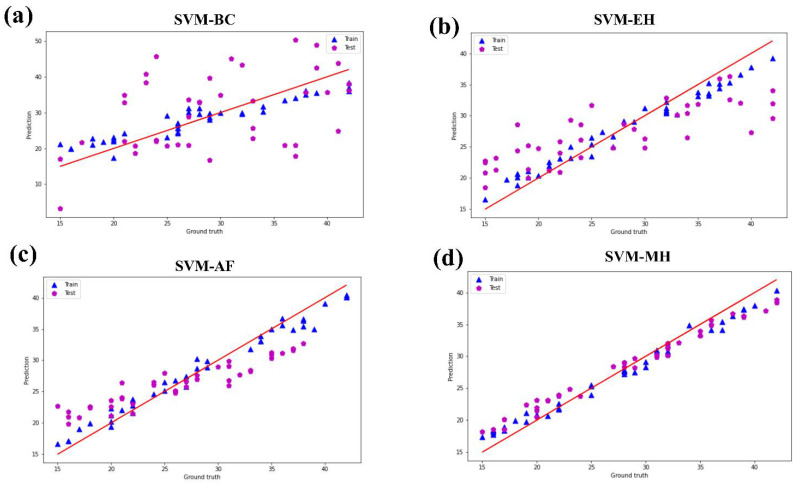

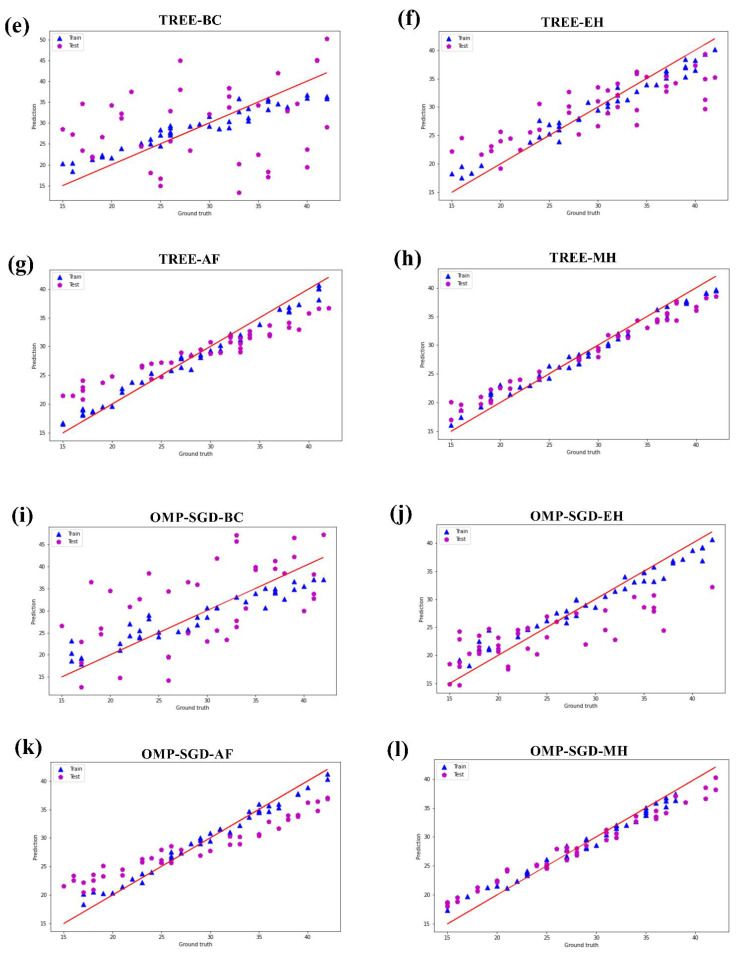

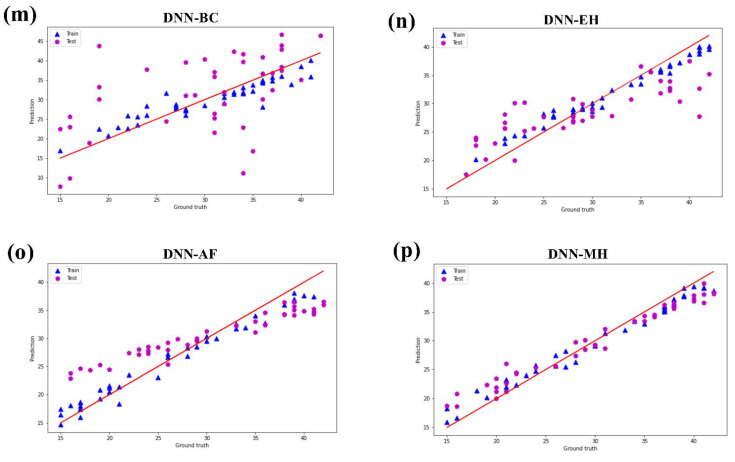
Training set and test set of overall preference for support vector machine (**a**–**d**), gradient boosted trees (**e**–**h**), orthogonal matching pursuit—stochastic gradient descent (**i**–**l**), and deep neural network (**m**–**p**). (**a**,**e**,**i**,**m**) are BC. (**b**,**f**,**j**,**n**) are EH. (**c**,**g**,**k**,**o**) are AF. (**d**,**h**,**l**,**p**) are MH.

Of the classification accuracy and regression of machine learning algorithms for predicting overall preferences, DNN is the highest (0.983 and 0.966), OMP-SGD is the second (0.929 and 0.913), and SVM and GDBT are the last. Table 4 shows the overall preference for each cooking method under each machine learning algorithm. Figure 2 shows that all algorithms are in good agreement with experimental data considering the training set, test set, and considering the nonlinear characteristics of sensory analysis. However, considering scrutiny, OMP-SGD is in better agreement with DNN, and AF is in better agreement with MH.

**Figure 3:**
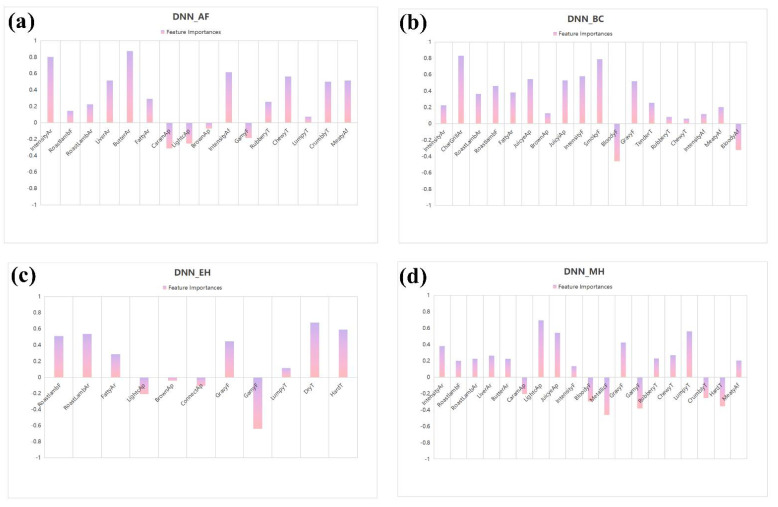

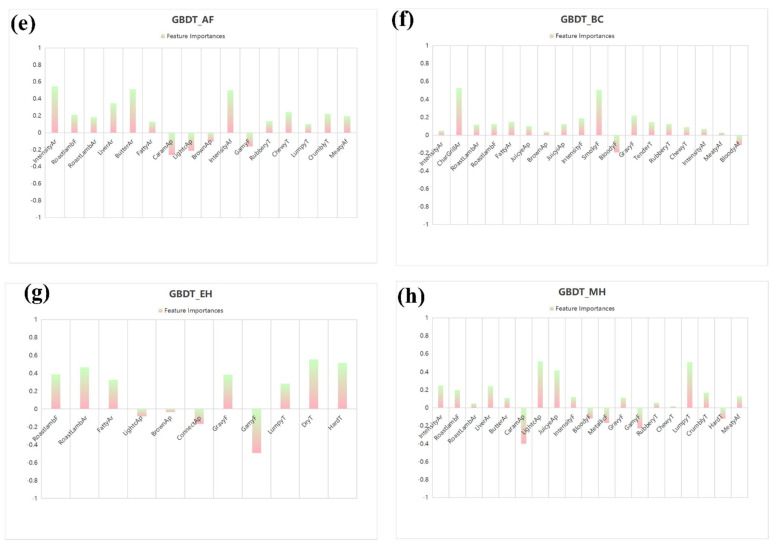

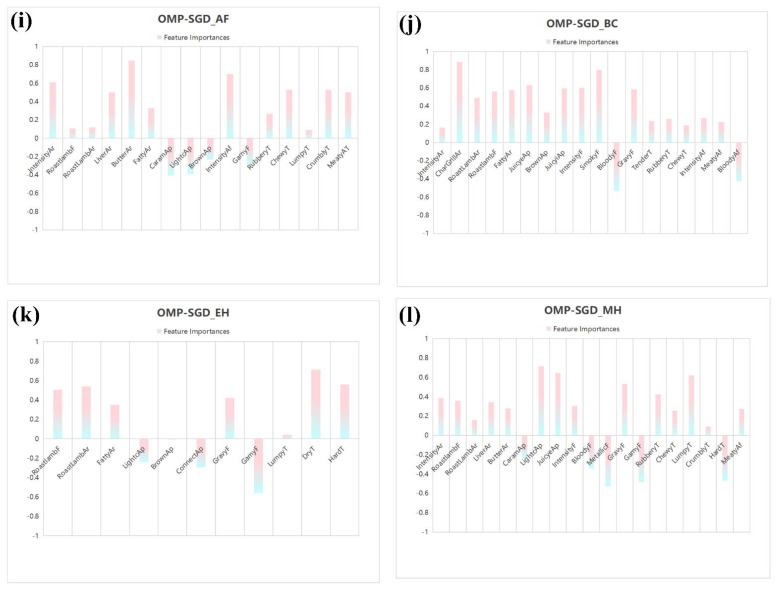

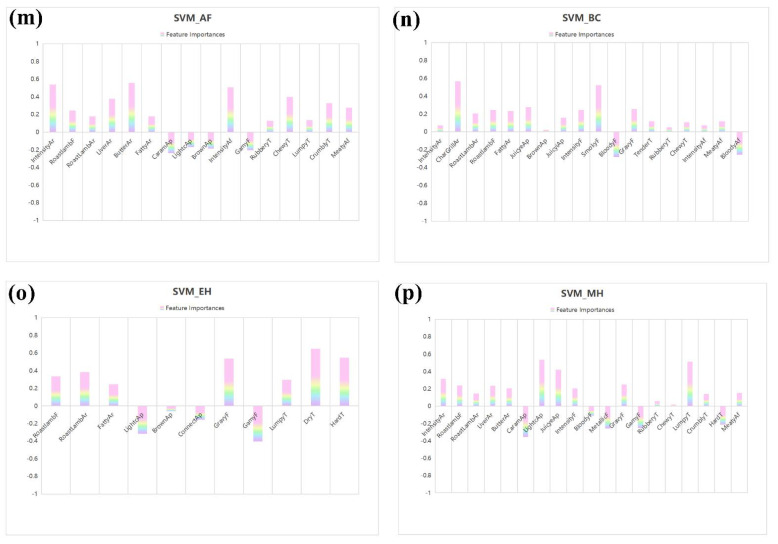
Important variables of the machine learning methods deep neural network (**a**–**d**), gradient boosted trees (**e**–**h**), orthogonal matching pursuit—stochastic gradient descent (**i**–**l**), and support vector machine (**m**–**p**).

Figure 3 exhibits the variables of importance for overall liking, considering a relative scale ranged from −1 to 1, representing the consumer preferences (overall liking score) of lamb shashliks with four different cooking methods (BC, AF, EH, and MH). Correlation coefficients above 0.5 were considered to be large effect size (Cohen, 1992). It is probable to see that the samples with BC in the DNN model were associated with the attributes char-grilled aroma, smoky flavor, juicy external appearance, juicy internal appearance, intensity flavor, and gravy flavor. BC in the OMP-SGD model was associated with the attributes char-grilled aroma, smoky flavor, fatty flavor, roast lamb flavor, juicy external appearance, juicy internal appearance, intensity flavor, and gravy flavor. BC in the SVM and GDBT models were both associated with attributes char-grilled aroma and smoky flavor. For AF, the attributes of samples in the DNN and OMP-SGD models were associated with attributes intensity aroma, butter aroma, intensity aftertaste, meaty aftertaste, chewy texture, lumpy on chewing texture, and liver aroma, while the attributes in both the SVM and GDBT models were associated with intensity aroma, butter aroma, and intensity aftertaste. For EH, the samples were associated with the attributes roast lamb flavor, roast lamb aroma, dry texture, and hard texture in the DNN and OMP-SGD models and were associated with the attributes dry texture and hard texture in the SVM and GDBT models. The MH samples in the DNN model were associated with the attributes light color external appearance, juicy external appearance, and lumpy on chewing texture. The MH samples in the OMP-SGD model were associated with the attributes light color external appearance, juicy external appearance, gravy flavor, and lumpy on chewing texture. The MH samples in the SVM and GDBT models were both associated with the attributes light color external appearance and lumpy on chewing texture.

Overall, the attributes char-grilled aroma and smoky flavor were the most important variables for all machine learning methods in BC, the attributes butter aroma, intensity aroma, and intensity aftertaste were the most important variables for all machine learning methods in the AF samples, the attributes dry texture and hard texture were the most important variables for all machine learning methods in the EH samples, and the attributes light color external appearance and lumpy on chewing texture were the most important variables for all machine learning methods in the MH samples.

The attributes of the four cooking methods of lamb shashliks are classified and predicted by machine learning, as shown in Table 5, and the accuracy rates are all higher than 0.95. Therefore, the sensory attributes of each of the above-mentioned products can represent the corresponding products.

### 3.4. Qualitative Research

It was found that the buttery aroma can be perceived by Generation Z consumers through the CATA methodology. According to the results that the second dimension of CA is closely related to butter aroma and butter aroma is an important variable attribute of AF, the interviews were conducted. The best model (Table 5), determined by the cross-validation statistics of the interaction model, included factors A5, B1, and C2. The balanced accuracy of the training set was 0.87, and the consistency ratio of the ten-fold cross-validation of the balance accuracy of the test set was 10:10, which means that the result is significant in 10 of the 10 cross-validations. Meanwhile, the model was significantly better than the other models (*p* < 0.05). 

Twenty-nine Gen Z consumers were interviewed. The 25th interview elicited no new coding content, but it was necessary to interview a further four participants to verify that the data were saturated [50]. No new themes appeared in these additional interviews, indicating that the data reached saturation. The text data were entered step-by-step to generate nodes, including four first-level and eight second-level nodes (Table 6). 

**Figure 4:**
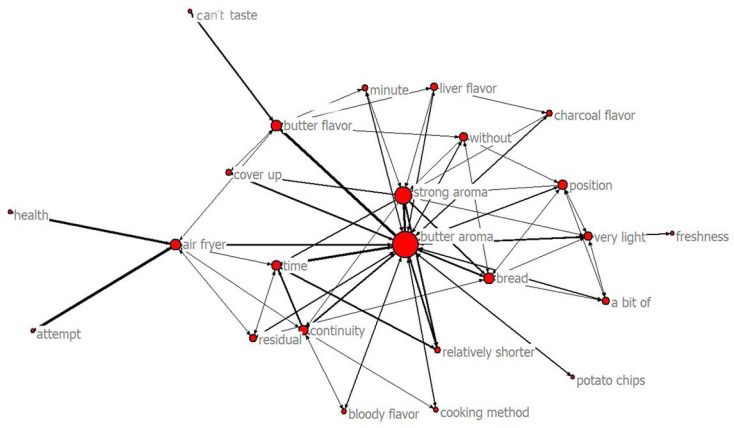
In the network diagram, nodes represent factors, and the connections between nodes represent the relationship between factors. The higher the centrality of the node degree, the more the point is in the center of the network. The thickness of the connection between nodes is positively correlated with the closeness of the relationship.

In the network diagram created by SNA (Figure 4), buttery aroma was the node with the highest centrality, indicating that it was the critical sensory attribute in this study. The thickness of the connection between nodes and multiple lines connecting the nodes is informative. For example: (i) Gen Z consumers who pursue “health” are willing to “attempt” the lamb shashliks made by the “air fryer” which exude a “strong” “buttery aroma” that changes with “time”; and (ii) lamb shashliks made by the “air fryer” have a “buttery aroma”, but consumers “can’t taste” a “buttery flavor”. SNA demonstrates that Gen Z consumers can recognize a buttery aroma.

## 4. Discussion

### 4.1. Application of Machine Learning

In fact, each machine learning method has advantages and disadvantages, reinforcing the need for using more than one method. Our findings reinforce the need for assessing different algorithms to evaluate the different performance, and to obtain only coincident responses, considering the different statistical modeling involved. It is worth noting that machine learning algorithms can be preferable to multiple regression and least partial squares because fewer assumptions are needed for the modeling to work well, in addition to that the assumptions of normality and absence of collinearity, normally present in sensory data, are ignored [51]. 

When evaluating a cooking method through four machine learning algorithms, it can be seen that the values of the respective algorithms are different, but the most important variables of sensory attributes in this product are similar. For lamb shashliks using BC, in the DNN model, the values of char-grilled aroma and smoky flavor were 0.83 and 0.79, respectively, while in the OMP-SGD model, the values of char-grilled aroma and smoky flavor were 0.89 and 0.80, respectively. Even in the SVM and GDBT models, the values of char-grilled aroma and smoky flavor were in the first two digits. Therefore, the most important variables for sensory attributes of lamb shashliks using BC are char-grilled aroma and smoky flavor. Similarly, the most important variables for the sensory attributes of AF lamb shashliks were butter aroma, intensity aroma, and intensity aftertaste, and the most important variables for sensory attributes of EH lamb shashliks were dry texture and hard texture. The most important variables for sensory attributes of MH lamb shashliks were light color external appearance and lumpy on chewing texture. It can be concluded that even if it is a lamb product, the important variables regarding sensory attributes would be different when processed with different cooking methods. This explains why the grilled lamb shashliks prepared using BC, AF, EH, and MH were easily distinguishable.

In addition, there were also some of the least important variables in the four products, such as caramel on bottom external appearance, gamy flavor, bloody flavor, metallic flavor, and bloody aftertaste. Gen Z consumers would be adversely affected by the least important variables, which would affect their product preferences. The gamy flavor of lamb results from several metabolites involved in energy production via the Krebs cycle and the Embden–Meyerhof–Parnas pathway [52]. Some consumers strongly dislike this particular flavor. The bloody flavor, metallic flavor, and bloody aftertaste were reminiscent of iron and metal, which are so strong that many people cannot accept it. Consumers’ appetite was reduced, and the texture and flavor of the products were changed by the caramel on bottom external appearance. In the process of making lamb shashliks, the generation of the least important variables should be avoided and the important variables of the products should be enhanced.

### 4.2. Identification of Sensory Attributes of Lamb Shashliks

The application of a 9-point hedonic scale allowed Gen Z consumers to discriminate between lamb shashliks prepared by different cooking methods. Significant differences in liking scores were found between samples for four different cooking methods (*p* < 0.05). As shown in Table 2, the overall liking scores of AF and BC samples are both greater than 6 points, and the overall liking scores of EH and MH samples are relatively low. As shown in Table 4, the accuracy of the four machine learning methods on consumers’ overall preference for the four different cooking methods of lamb shashliks is higher than 0.8 and most of them are higher than 0.9. Thus, it can be seen that consumers have a better identification for AF.

Regarding the four products of lamb shashliks, Gen Z consumers identified the sensory attributes of each grilled lamb shashlik. The dry texture as well as the hard texture can cause discomfort to consumers in the process of tasting the lamb shashliks. EH is associated with the sensory attributes that negatively affected their sensory evaluation, which may be the reason for the lower score, while the reason for the lower score of MH samples is associated with the light color external appearance, because in barbecue products the light color external appearance is not appetizing to consumers.

The sensory attributes characteristic of BC were char-grilled aroma and smoky flavor. The char-grilled aroma is the unique aroma produced by smoke from the burning charcoal being absorbed by the grilled lamb shashliks. The smoky flavor is produced by the formation of volatile organic compounds such as phenols, furans, and aldehydes [53]. According to informal interviews, smoky flavor and char-grilled aroma are the main reasons for Gen Z consumers to choose lamb shashliks prepared by BC. BC lamb shashliks have been handed down from ancient times as a traditional food, and their unique aroma and flavor are the biggest advantages. The lamb shashliks prepared by AF also have a unique aroma that is distinct from BC and can catch the attention of Gen Z consumers. As the most important variables, butter aroma and intensity aroma were readily perceived by Gen Z consumers in shashliks prepared by AF and were highly appreciated. However, they were not perceived in other cooking methods or had no noticeable effect on the evaluations. For example, in MH samples, some Gen Z consumers can only perceive a faint buttery aroma, while in BC and EH samples, no buttery aroma can be perceived.

This study adopted the grounded theory to explore whether there was a buttery aroma in the grilled lamb shashliks prepared by AF. First, according to the results that the second dimension of CA is strongly related to butter aroma and AF located at the second dimension was associated with buttery aroma, in-depth one-on-one interviews were carried out with the Gen Z consumers who perceived the buttery aroma (i) to set up guiding questions (Section 2.6.1), (ii) to collect data that affected the consumption experience, and (iii) to explore the general characteristics that affect the Gen Z consumer experience. The environmental interaction combination reclassifies the research subjects into “high-risk” and “low-risk” groups who can detect the aroma, and the estimated OR value of the risk interaction effect of the smell–risk interaction effect was 2.59. In this paper, the OR value greater than 1 indicated that the factor is an important factor. The “high-risk” group easily detected the taste, and the “low-risk” group had difficulty detecting the taste. The “high-risk” group was slightly older, had tasted AF food, was mostly male, and was 2.59 times higher than the normal group (Table 5). The ability of Gen Z consumers to smell the buttery aroma was closely related to their age, gender, and whether they had previously tasted food prepared in an air fryer.

Second, the nodes generated by the grounded theory are the extraction and refinement of the interview content. The intention was that consumers would describe the smell of the samples and the characteristics of the buttery aroma based on their own cognition. Consumers who could detect the buttery aroma were asked what the aroma was similar to (potato chips, bread, milky tea), what was its duration (tens of seconds, soon, a few minutes), and how intense it was (strength). When Gen Z consumers started eating, they perceived a buttery aroma that was associated with honey-flavored potato chips, smelled a sweet aroma, confirmed this repeatedly, and finally identified buttery aroma.

When MDR analysis was introduced, this study found that the statement “Gen Z consumers who can smell the buttery aroma” was not strictly accurate. It should be “Gen Z consumers who are sure to smell a certain aroma that is the buttery aroma”. They may not have a sensitive sense of smell, or it may even be poor (for example, due to rhinitis), and their perception has nothing to do with gender or region. It is simply that Gen Z consumers may have previously been exposed to food prepared in an air fryer, and their experience (due to age) is relatively rich, so they subconsciously like this aroma. For these consumers, this aroma is associated with products having a buttery aroma (potato chips, bread, milky tea) with which they are familiar, thereby confirming that grilled lamb shashliks prepared by AF have a buttery aroma. Gen Z consumers who do not possess these two characteristics have poor association ability and have difficulty in quickly associating the aroma with a product, so they automatically ignore this aroma and judge which ones are more familiar or stronger. Thus, the judgment process for the buttery aroma tends to be a psychological process of self-improvement, self-denial, and remodeling. 

Finally, SNA was applied to verify the relationship between Gen Z consumers who smelled the buttery aroma, and the cognitive process by creating a visual network structure (Figure 4). This showed that the significant feature of AF was buttery aroma. In summary, using CATA methodology and grounded theory, the process of smelling a buttery aroma was described, proving that cognitive methods can identify this aroma, which is helpful for consumers to improve their descriptions and for determining the tastes experienced by consumers. 

In short, the AF lamb shashlik is a novel product, with outstanding sensory attributes for Gen Z consumers, and is characterized as healthy and fast. This novel product may be further optimized to improve its potential in the food market sector.

## 5. Conclusions

This study was designed to develop novel lamb shashliks for Gen Z consumers, which adopt the CATA methodology and machine learning algorithms. It was concluded that lamb shashliks using the AF technique were preferred by Gen Z consumers, because AF lamb shashliks have the unique attribute buttery aroma. Grounded theory and the social network analysis (SNA) method were utilized to explore why consumers chose AF, demonstrating that Gen Z consumers who had previously tasted AF lamb shashliks could easily perceive the buttery aroma. Gen Z consumers have a higher overall preference for grilled lamb shashliks prepared by AF—a novel product with the potential to replace BC. The research results can provide the basis for the study of Gen Z food consumerism lifestyle and determine the sensory factors that can enhance customer preference, which is very important for the successful operation of the food industry.

## Figures and Tables

**Table 1:** Frequency of selection of the CATA attributes for the four samples.

Attributes		BC	AF	EH	MH
Aroma	Intensity aroma ***^1^	48	77	35	26
	Char grilled aroma ***	102	45	51	18
	Roast lamb aroma ***	96	90	90	65
	Liver aroma ***	20	67	31	47
	Buttery aroma***	13	105	39	45
	Oily aroma ^ns^	44	56	58	48
	Fatty aroma ***	82	67	57	43
Appearance	Caramel on bottom external appearance ***	60	64	58	34
	Light color external appearance ***	47	30	48	87
	Dark external appearance ^ns^	68	86	73	43
	Juicy external appearance ***	78	32	39	40
	Pink internal appearance ^ns^	27	16	24	13
	Brown internal appearance ***	91	82	73	52
	Juicy internal appearance ***	46	30	19	22
	Connective tissue internal appearance ***	20	26	25	26
	Wet external appearance ^ns^	54	40	37	54
	Greasy external appearance ^ns^	59	28	43	42
Flavor	Intensity flavor ***^2^	50	27	35	25
	Smoky flavor ***	113	23	37	11
	Roast lamb flavor ***	114	97	90	61
	Liver flavor ^ns^	16	72	45	66
	Bloody flavor ***	43	33	50	39
	Metallic flavor ***	78	46	33	50
	Fatty flavor ***	21	22	57	26
	**Gam** ey flavor ***	60	66	79	64
	Greasy flavor ^ns^	38	30	39	20
	Gravy flavor ***	39	64	58	41
	Bitter flavor ^ns^	20	16	23	17
	Sour flavor ^ns^	34	36	31	39
	Sweet flavor ^ns^	14	23	18	11
Texture	Tenderness texture **	41	40	30	39
	Rubbery texture ***	62	40	43	46
	Chewy texture ***	68	86	65	53
	Lumpy on chewing texture ***	23	38	44	53
	Crumbly texture ***	44	50	27	45
	Spongy texture ^ns^	12	15	23	26
	Dry texture *	55	63	72	59
	Hard texture ***	42	56	59	53
Aftertaste	Intensity aftertaste *** ^3^	25	37	22	28
	Meaty aftertaste ***	79	82	72	53
	Liver aftertaste ^ns^	12	43	28	41
	Bloody aftertaste *	40	29	35	32
	Oily aftertaste ^ns^	45	45	51	43
	Lactic aftertaste ^ns^	13	13	16	30
	Sour aftertaste ^ns^	17	31	32	24
	Sweet aftertaste ^ns^	8	22	12	17

List of sensory attributes used in the CATA ballot (*p*-value below 0.050 on Cochran’s Q test is represented in bold) and Cochran’s Q test was used to detect significant differences between attributes. *** Indicates significant differences among samples at *p* ≤ 0.001. ** Indicates significant differences at *p* ≤ 0.01. * Indicates significant differences at *p* ≤ 0.05. ^ns^ Indicates no significant differences (*p* > 0.05). ^1^ Means first impact of aroma strength in the nose. ^2^ Means first impact of flavor strength in the mouth. ^3^ Means first impact of aftertaste strength staying in the mouth.

**Table 2:** Mean overall liking, based on 9-point hedonic scale, (±SD) according to the type of grilled lamb shashlik for different cooking methods. F-values from the one-way ANOVA, with different cooking methods as factors on overall liking.

Cooking Methods	Mean (±SD)	F *p*
BC	6.91 (± 1.65)	F = 181.514 *p* < 0.001
AF	6.64 (± 1.66)	
EH	5.23 (± 1.83)	
MH	4.65 (± 1.93)	

**Table 3:** LSD (Least Significant Difference) test, after one-way ANOVA, the average value was compared.

Control Product	Product	Mean Value Difference (I–J)	Significance	95% Confidence Interval	
				Lower Limit	Upper Limit
AF	BC	−0.263 *	0.022	−0.49	−0.04
	EH	1.410 *	0	1.19	1.63
	MH	1.992 *	0	1.77	2.22
BC	AF	0.263 *	0.022	0.04	0.49
	EH	1.673 *	0	1.45	1.9
	MH	2.254 *	0	2.03	2.48
EH	AF	−1.410 *	0	−1.63	−1.19
	BC	−1.673 *	0	−1.9	−1.45
	MH	0.581 *	0	0.36	0.81
MH	AF	−1.992 *	0	−2.22	−1.77
	BC	−2.254 *	0	−2.48	−2.03
	EH	−0.581 *	0	−0.81	−0.36

* Indicates that the significance level of the mean difference is 0.05.

**Table 4:** Using machine learning methods to classify and predict the accuracy of consumers’ overall preference and lamb shashliks preference for four various cooking methods.

Overall Preference				
	BC	AF	EH	MH
DNN	0.9152	0.9575	0.957	0.9543
OMP-SGD	0.9082	0.9596	0.9567	0.9567
SVM	0.9798	0.9184	0.9444	0.912
GDBT	0.8878	0.9378	0.8275	0.9311
Lamb shashliks of preference				
	BC	AF	EH	MH
DNN	0.9807	0.9823	0.9780	0.9698
OMP-SGD	0.9819	0.9874	0.9776	0.9775
SVM	0.9761	0.9763	0.9775	0.9678
GDBT	0.9584	0.9678	0.9275	0.9810

**Table 5:** Determination of the best model of MDR, a total of 11 variables and 660 recorded data points were screened out according to the basic information of the interviewees and imported into the MDR software, after fitting all the models in the 1–3 interaction order.

Interaction Order	Factors Included in the Model	Training Set Balance Accuracy	Test Set Balance Accuracy	Cross-Validation Consistency Rate (Ratio)	Odds Ratio (OR) Value	*p*-Value
1	A5 (Have you tasted air fryer food)	0.56	0.50	8:10	1.55	0.04
2	A5 (Have you tasted air fryer food), B1 (Age)	0.61	0.58	9:10	0.88	0.97
3	A5 (Have you tasted air fryer food), B1 (Age) C2 (gender)	0.87	0.86	10:10	2.59	0.03

**Table 6:** Node level and material information of SNA.

Primary Node	Secondary Node	Reference Node Example
Cognition of buttery aroma	Comparison with products with a similar aroma	The buttery aroma is similar to that of Lay’s honey potato chips.
	Cognition of duration and intensity	Compared with the aroma of dairy products, it is not as strong as dairy products. The duration of milk aroma is short.
Cognition of AF	Cognition of roasting process	The roasting speed is fast. The strong aroma can be smelled in a few minutes. It is mixed with a light milky aroma, but it dissipates quickly.
	Cognition of comparison with other roasting methods	It is healthy and suitable for a fast-paced life. It tastes more delicious than EH and MH.
Self-awareness	describe the self-awareness process and experience	Not affected by the surroundings, I smelled the buttery aroma. Although I have rhinitis, it does not affect my smell.
	Try to recognize the reason why you can smell the buttery aroma of AF	I think other cooking methods may have a buttery aroma, such as BC, which is just covered by the char-grilled aroma. The EH is a relatively dry texture, and the other aroma also covers the buttery aroma. The AF can more purely restore the taste of the ingredients. It instantly releases a lot of aromatic substances. Maybe I prefer sweets and am more sensitive to the buttery aroma, so the perception priority is higher. However, the milk aroma substances are volatilized afterward, which may be related to the roasted part.
Cognition of the future	What are expectations for AF?	It is more delicious.
	What are your expectations for the future development of barbecue	It is healthier and tastes better.

## Data Availability

Data is contained within the article.
